# Differential Go/NoGo Activity in Both Contingent Negative Variation and Spectral Power

**DOI:** 10.1371/journal.pone.0048504

**Published:** 2012-10-31

**Authors:** Ingrid Funderud, Magnus Lindgren, Marianne Løvstad, Tor Endestad, Bradley Voytek, Robert T. Knight, Anne-Kristin Solbakk

**Affiliations:** 1 Department of Neuropsychiatry and Psychosomatic Medicine, Division of Surgery and Clinical Neuroscience, Oslo University Hospital - Rikshospitalet, Oslo, Norway; 2 Department of Psychology, Lund University, Lund, Sweden; 3 Sunnaas Rehabilitation Hospital, Nesodden, Norway; 4 Department of Psychology, University of Oslo, Oslo, Norway; 5 Department of Neurology, University of California San Francisco, San Francisco, California, United States of America; 6 Helen Wills Neuroscience Institute, University of California, Berkeley, California, United States of America; 7 Department of Psychology, University of California, Berkeley, California, United States of America; University of British Columbia, Canada

## Abstract

We investigated whether both the contingent negative variation (CNV), an event-related potential index of preparatory brain activity, and event-related oscillatory EEG activity differentiated Go and NoGo trials in a delayed response task. CNV and spectral power (4–100 Hz) were calculated from EEG activity in the preparatory interval in 16 healthy adult participants. As previously reported, CNV amplitudes were higher in Go compared to NoGo trials. In addition, event-related spectral power of the Go condition was reduced in the theta to low gamma range compared to the NoGo condition, confirming that preparing to respond is associated with modulation of event-related spectral activity as well as the CNV. Altogether, the impact of the experimental manipulation on both slow event-related potentials and oscillatory EEG activity may reflect coordinated dynamic changes in the excitability of distributed neural networks involved in preparation.

## Introduction

Preparatory brain activity is a critical precursor for successful execution of goal-directed behavior. Preparation increases cognitive and behavioral efficiency due to pre-activation of the sensory and motor cortices needed to execute an appropriate response at the correct time. This pre-activation is proposed to be controlled by top-down signals from prefrontal cortical areas [1], [2].

The most commonly studied electroencephalographic (EEG) marker of preparatory activity is the contingent negative variation (CNV) event-related potential (ERP) [3]. The CNV reflects a tonic modulation of the EEG signal in the preparatory period between a warning (S1) and an imperative (S2) stimulus. With sufficiently long (e.g. 3–4 sec) inter-stimulus intervals two components can be distinguished; an early CNV and a late CNV [4]–[6]. The early CNV has a frontal scalp distribution and is believed to index both an orienting response to S1 [7], and stimulus processing or evaluation of the cognitive information contained in S1 [2], [8]. The late CNV is considered to be an index of anticipatory attention for the upcoming stimulus and motor preparation needed to respond [9], [10]. In line with this the late CNV is thought to be a combination of at least two slow waves, a movement preceding negativity (MPN) and a stimulus preceding negativity (SPN) [11].

ERPs do not encompass all the electrophysiological changes related to an event. Changes in magnitude or phase of the different frequency bands composing the EEG are also associated with stimulus and cognitive processing. Some of the modulations of the frequency bands are also reflected in the ERP [12], [13], but many are averaged out during data processing. Investigations of oscillatory activity in the S1–S2 interval can therefore provide additional information about the neurophysiological responses associated with preparatory processes. Indeed, Babiloni et al. (1999) showed that alpha event-related desynchronization (ERD) preceding self-initiated movements have more widespread cortical sources than movement-related potentials (MRPs). The authors suggested that whereas the MRPs may reflect specific processes such as selection and running of task-specific motor commands, alpha ERD could reflect the functional alerting of wider cortical neural populations [14]. A number of studies have shown prestimulus ERD. Cued movements, such as responses to imperative stimuli in a CNV paradigm, are preceded by a reduction of alpha power [15]–[17]. In these studies alpha ERD can reflect both anticipation for the imperative stimulus and preparation for the response. Other studies show that movements that are not a response to a stimulus, such as voluntary movements [18]–[20], and stimuli that do not require a motor response, are also preceded by alpha and/or beta ERD [21], [22].

The above studies investigated alpha and/or beta activity exclusively. The different spectral bands have been linked to partly separate and partly overlapping cognitive, perceptual and sensory functions [23]. For example, alpha and beta have not only been associated with motor and sensory preparation, but also with attention, as has also the gamma band. Further, theta and alpha have been associated with for example working memory processes, beta with sensorimotor integration and low gamma (30–50 Hz) with integration of sensory information [for review, see 24, 25]. Accordingly, extending the EEG frequency analysis to other spectral bands has the potential to provide insight into other aspects of information processing compared to when only the alpha or beta band is examined. Few studies have investigated preparatory activity in multiple bands. Gomez and colleagues reported a reduction of power in a broad spectral band, ranging from delta to low gamma [26].

It is necessary to compare trials calling for motor preparation to trials where no such preparation is required to investigate the specific relation of the CNV and of spectral power reduction to motor preparation. ERPs and concomitant event-related oscillatory activity have not been systematically studied in CNV paradigms where S1 contains Go versus NoGo information. The CNV is typically increased in conditions requiring a response to S2 versus a condition not requiring a response to S2 in Go/NoGo designs [27], [28]. Thus, a call for both motor preparation and stimulus anticipation generates a CNV of larger amplitude compared to a call for stimulus anticipation alone. As discussed above, both motor preparation and anticipation for a stimulus is accompanied by alpha/beta ERD. Then, will also alpha/beta ERD be larger in trials where both motor preparation and stimulus anticipation is needed versus trials where only stimulus anticipation is involved? Such a finding would imply larger alpha/beta ERD when a greater degree of preparatory processes is required.

Two studies have investigated spectral EEG activity in a Go/NoGo CNV paradigm, and they reported divergent results. Filipovic et al. found the expected Go/NoGo difference in CNV amplitude, but alpha power did not vary significantly between conditions [29]. In contrast, Babiloni et al. reported that alpha power was reduced in the Go compared to the NoGo condition [30]. In the latter study Go and NoGo conditions were presented in separate recording blocks. Importantly, in both studies the Go condition did not consistently involve motor preparation, but rather preparing for either a Go or NoGo signal. Consequently, electrophysiological processes related to preparing versus not preparing for a response were not fully separated.

Filipovic suggested that their results indicate that there is no direct coupling between the CNV and alpha ERD. This view is supported by other studies [14], [15], [31], [32]. Nevertheless, the literature also points to a relationship between negative slow waves and alpha power decrease and both measures are thought to reflect increased cortical activation [33], [34]. In support of this, visual working memory research has demonstrated that sustained visual cortical negativity scales with working memory load [35] and that this negativity may arise due to asymmetric alpha amplitude modulations [36]. Further evidence for a role of alpha reduction in cortical excitability comes from patients with unilateral prefrontal lesions who show enhanced alpha in the ipsilesional visual cortex [37] as well as attenuated visual cortical ERP negativity in the affected hemisphere [38]. Finally, the notion of low band spectral decrease as a metric of cortical activity is supported by the finding that narrow band local field potential oscillations below 30 Hz are negatively correlated with neuronal spiking [39]. Taken together, this evidence suggests that a sustained negativity such as the CNV may reflect cortical excitation during movement preparation and that this, in turn, may be reflected by an alpha power decrease. Further, a reduction of CNV amplitude in trials where movement preparation is not required should be accompanied by less alpha power reduction than for Go trials. We suggest that the reason why an alpha power Go/NoGo difference was not consistently found in the two studies investigating preparatory Go/NoGo spectral activity could be that Go and NoGo trials were not sufficiently different. We hypothesize that increasing the differences will result in alpha power differentiation of the two trial types.

The aim of the present study was twofold. First, we examined whether preparatory alpha activity differs between Go and NoGo trials. This has not been investigated previously in a CNV paradigm where Go and NoGo conditions are fully distinct regarding motor preparation. To address this issue we designed an experiment where S1 provided definitive Go or NoGo information. In addition to the CNV, spectral activity was investigated in the same interval (400 ms post S1 to 50 ms pre S2) using the Event-Related Spectral Perturbation (ERSP) measuring event-related dynamics of the EEG spectrum [12]. We expected to confirm that S1 Go signals evoke a CNV of larger amplitude than S1 NoGo signals.

We hypothesized that when Go and NoGo conditions are distinct, the differential CNV amplitude effects observed to these trial types would be accompanied by Go/NoGo differences in alpha activity. Specifically, we postulated that CNV increases in Go trials would be associated with reduced alpha band activity. Also, to the extent that CNV amplitudes are reduced in NoGo compared to Go trials, there will be less alpha power decrease in NoGo trials.

Our second aim was to examine whether the hypothesized Go/NoGo difference in alpha power was accompanied by a similar modulation of other EEG frequency bands. A broadband reduction of power has been demonstrated for Go trials in one previous study [26], but the specificity of such a broadband power reduction to Go trials has not been investigated in a simple Go/NoGo CNV paradigm. We examined spectral activity from theta to gamma. Although gamma activity above 50 Hz recorded with scalp EEG is severely attenuated due in part to the 1/f frequency power fall-off [40], we explored spectral oscillations up to 100 Hz. We hypothesized that not only alpha, but also spectral bands up to at least lower parts of the gamma range would show a Go/NoGo difference of event-related power in the S1–S2 interval.

## Methods

### Participants

Eighteen healthy persons participated and two subjects were excluded due to excessive EEG artifacts. Six of the remaining 16 individuals were females. Mean age was 42.6 (SD 12.2) and mean years of education was 13.2 (SD 2.5). All had above-average IQ (mean 114.4, SD 7.4) as measured with the Wechsler Abbreviated Scale of Intelligence (Wechsler, 1999).

### Ethics Statement

All participants provided written, informed consent to take part in the study and were recompensed. The study was approved by the Regional Committee for Medical Research Ethics, Region South Norway and was conducted in agreement with the Helsinki declaration.

### Experimental Task

Participants were seated 1 meter from a computer screen. A tone of 250 ms duration constituted S1, with the Go-signal differing from NoGo in pitch (1500 vs. 1000 Hz, respectively). Following a 3500 ms S1-offset to S2-onset delay, a white circle (S2; 250 ms) was centrally presented. Participants were instructed to press a button as quickly as possible when S2 followed an S1 Go-signal, and not to press after an S1 NoGo-signal. The circle contained a black arrow pointing to the right or the left. A right hand button press was required to a right-pointing arrow, and a left hand button press to a left-pointing arrow. Visual feedback concerning accuracy and reaction time (RT) was delivered 3500 ms after S2. Intertrial-interval (offset feedback to onset S1) varied randomly between 1000 and 2500 ms. Stimuli were presented in two blocks, each consisting of 30 Go- and 15 NoGo-trials randomly presented. Both blocks and trial types contained 50% left pointing and 50% right pointing S2-arrows. A training session including eight Go- and four NoGo-trials was conducted before EEG recording. Stimulus presentations and response recordings were controlled using E-prime software, version 2.0 (Psychology Software Tools, Pittsburgh, PA).

### EEG Recording and Analyses

EEG-data were acquired using a 128-channel HydroCel Geodesic Sensor Net and Net Amps 300 amplifier (Electrical Geodesics, Eugene, OR) with a 250 Hz sampling rate, a 24 bit analog-to-digital converter and a DC to 125 Hz bandpass. Impedance was generally maintained below 50 kΩ, with 100 kΩ as an upper limit [41]. A Cz reference was used during recording.

Continuous EEG data were high-pass filtered offline using Matlab: The signal was first filtered with a 0.05 Hz low-pass FIR filter (roll off 0.001 to 0.05 Hz, Equiripple, 8 dB attenuation in the stop band). The resulting low-passed signal was subsequently subtracted from the unfiltered signal, thus resulting in 0.05 Hz high-pass filtered data. All subsequent analyses were performed using custom-written scripts in MATLAB (Natick, MA) based on EEGLAB [42] functions. Bad channels were identified through visual inspection and interpolated. Mean number of interpolated channels was 8 (±3 SD). Ocular artifacts were removed using independent components analysis. The data were rereferenced to average reference with ocular channels excluded.

#### ERP

Continuous EEG data were epoched time-locked to S1 onset from −1000 to 4700 ms, with −500 ms to 0 as baseline. Trials with incorrect responses and/or amplitude values exceeding ±150 µV were rejected, leaving a mean number of trials in individual average files of 52 (±5.2 SD) for Go and 24 (±4.4 SD) for the NoGo condition.

#### ERSP

Continuous EEG data were band passed into theta (4–7 Hz), low alpha (8–10 Hz), high alpha (11–13 Hz), low beta (14–20 Hz) and high beta (21–30 Hz) frequency bands, as well as two low gamma (31–47 and 53–80 Hz) and one high gamma (81–100 Hz) band. We chose these bands *a priori* to allow for comparisons to the existing literature as well as to minimize the need for correcting for multiple comparisons across the entire time-frequency spectrum. Bandpass filtering was performed via point-by-point multiplication of a Gaussian with the fast Fourier Transform of the continuous EEG. The Gaussian standard deviation was 10% of the center frequency resulting in full width at half maximum of 0.2355 of the center frequency. The analytic amplitude (absolute value of the Hilbert transform) for each passband was used to create a grand average time-frequency event-related potential. These ERSPs were segmented time-locked to onset of S1 from −1000 to 4700 ms, with the 1000 ms pre-S1 window serving as baseline. Trials removed in the ERP analysis were also removed from the ERSPs, rendering the two datasets comparable.

### Statistical Analysis

**Figure 1:**
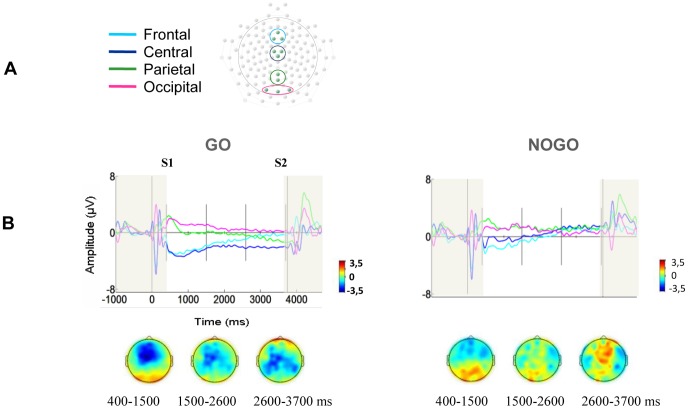
Contingent negative variation in Go and NoGo conditions. (**A**) Electrode layout with regions of interest. (**B**) The contingent negative variation is larger in the Go compared to the NoGo condition over frontal, central and parietal regions of interest. The colors of the ERPs illustrate the region of interest, indicated in (A), that each ERP represents. The ERPs are 7 Hz low-pass filtered for illustration purposes. Scalp topographies represent average activity within 3 post S1 time intervals indicated with vertical lines in the time-power plots. Intervals not included in the analysis are shaded.

Regions of interest (ROI) electrode groups were established over 4 midline sites: frontal, central, parietal and occipital (Fig.1A). These specific ROIs were chosen to allow for comparison to existing literature [i.e. 26, 30]. As the subject could not know until presentation of S2 what hand to respond with, we did not expect lateralized preparatory motor area activation. Thus, lateral electrode groups were not included. Statistical analyses were performed on mean values over electrodes within each ROI. For CNV, mean amplitudes from three time windows (400–1400, 1500–2600, and 2600–3700 ms post S1-onset) were extracted. These windows were chosen to cover the whole S1–S2 interval and to ensure that the first time window reflected the early CNV. For the alpha, beta and gamma ERSPs each of the three time windows were divided in two, resulting in six time windows being extracted from the ERSPs (400–950, 950–1500, 1500–2050, 2050–2600, 2600–3150, 3150–3700 ms post S1-onset). This was done to capture the faster changes of the ERSPs than that of the CNV in the 400 to 1500 ms time window. Considering the slow oscillations of theta, the same time windows as for the ERPs were extracted from the ERSPs of this frequency band. To investigate manual reaction time (RT) in successful Go trials, the median of each individual’s RTs to correct Go trials was computed. The mean of these individual median RTs was then computed.

ERSPs from each spectral band and the ERP data were subjected to repeated measures analyses of variance (ANOVAs) with time (6 or 3 intervals), topographical plane (4 ROIs) and condition (Go vs. NoGo) as within-subject factors. SPSS 18 for Windows (SPSS Inc.) was used for statistical analyses. For computations involving more than one degree of freedom, Greenhouse-Geisser epsilon (ε) and corrected p-values along with uncorrected degrees of freedom are reported. Effects involving differences between task conditions were of primary interest. Interactions involving condition resulted in planned contrast tests. Alpha was set to.05.

The relationship between Go-trial ERP and ERSP amplitude over the frontal and central regions was investigated using Pearson product moment correlation. ERP amplitudes in the early time window were correlated to ERSP power in the first time widow for theta and the first two time windows for alpha, beta and gamma. ERP amplitude in the middle time window was correlated with ERSP power in the middle time window for theta and the middle two time windows for alpha, beta and gamma. Finally, ERP amplitudes in the last time window were correlated to ERSP power in the last time window for theta and the last two time windows for alpha, beta and gamma. This resulted in 6 Pearson correlations per ROI for each band. Because of the multiple correlation analyses, alpha was set to.01.

## Results

### Behavioral Performance

Participants had a high hit rate to S2 in Go trials (99.2% (SD 1.1)) and made few commission errors to S2 in NoGo trials (3.8% (SD 4.4)). Mean RT to S2 in Go trials was 439 (SD 69) ms.

### Go/NoGo ERP

Visual inspection of the ERPs (Figure 1) suggested a robust CNV in Go compared to NoGo trials and the scalp topographies support a differential scalp distribution for the two conditions.

Statistical analysis confirmed an overall main effect of Condition (F(1,15) = 37.53, p<.001, η^2^ = .714). Significant interactions between Condition and Plane (F(3,45) = 5.11, p = .012, η^2^ = .254, ε = .675) and Condition and Time (F(2,30) = 6.64, p = .008, η^2^ = .307, ε = .811) reflected that the Go/NoGo difference was only significant over frontal, central and parietal ROIs (ps<.028), and that in the parietal ROI the Go/NoGo difference was only significant in the last time window (p = .012). Main effects of Plane (F(3,45) = 5.49, p = .013, η^2^ = .268, ε = .571) and Time (F(2,30) = 4.36, p = .033, η^2^ = .225, ε = .768) were modified by a Time x Plane interaction (F(6,90) = 7.28, p = .006, η^2^ = .327, ε = .253), as the scalp distribution of the CNV varied across time irrespective of condition.

### Go/NoGo ERSP

**Figure 2:**
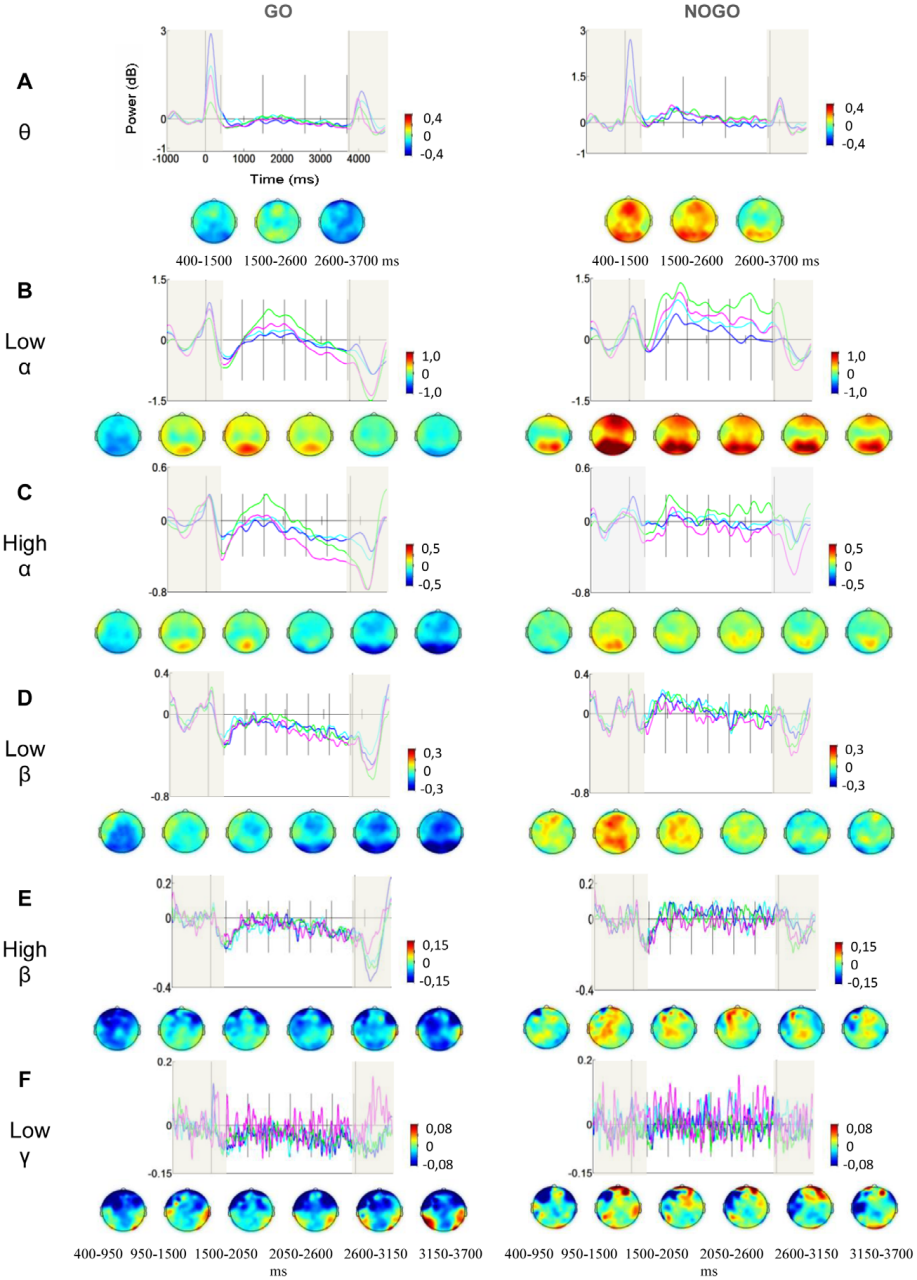
Go and NoGo activity for Event Related Spectral Perturbations. (**A–F**) Go and NoGo activity for theta, low alpha, high alpha, low beta, high beta and gamma. For all bands there is a Go/NoGo difference in power in the S1–S2 interval, with reduced power in the Go compared to the NoGo condition. The colors of the ERSPs indicate the region of interest that each ERSP represents; frontal (cyan), central (blue), parietal (green) and occipital (magenta) (illustrated in figure 1A). Theta scalp topographies represent average activity within 3 post S1 time intervals; alpha, beta and gamma topographies within 6 intervals, indicated with vertical lines in the time-power plots. Intervals not included in the analysis are shaded.

Visual inspection of the scalp topographies and time–power plots (Figure 2 and S1) suggests that while Go trials predominantly displayed reduced power compared to baseline, NoGo trials showed a tendency for increased power in theta to low gamma.

Statistical analysis confirmed a significant power difference between Go and NoGo trials for all bands except 81–100 Hz gamma. For theta, low alpha, both beta bands, and 31–47 Hz gamma there was a main effect of Condition (theta: F (1, 15) = 6.02, p = .027, η^2^ = .29; low alpha: F (1, 15) = 5.18, p = .038, η^2^ = .277; low beta: F (1, 15) = 26.43, p<.001, η^2^ = .59; high beta: F (1, 15) = 15.06, p = .007, η^2^ = .40; 31–47 Hz gamma: F (1, 15) = 5.96, p = .027, η^2^ = .28) that reflected lower event-related power in Go compared to NoGo conditions. For high alpha there was a trend towards a main effect of Condition (high alpha: F (1, 15) = 3.59, p = .077 η^2^ = .193). Post hoc analysis due to a Time x Plane x Condition interaction (F (15, 225) = 3.65, p = .013, η^2^ = .20, ε = .24) revealed that for high alpha the Go/NoGo difference was significant across Planes in the last two time intervals (p = .030 and.007), but not in the first four intervals (ps>.123). Post hoc analysis of 53–80 Hz gamma, due to a significant interaction of Plane and Condition (F (1, 15) = 5.22, p = .010, η^2^ = .26, ε = .71), revealed a significant difference between conditions over the occipital ROI (p = .032) that reflected higher power in the Go compared to the NoGo condition. However, visual inspection of individual topoplots and continuous EEG suggested that muscle artifacts in three participants were responsible for this isolated occipital 53–80 Hz gamma activity.

A main effect of Time was found for low (F (5, 75) = 6.49, p = .008, η^2^ = .30, ε = .33) and high alpha (F (5, 75) = 8.42, p<.001, η^2^ = .36, ε = .62) and for low (F (5, 75) = 6.78, p<.001, η^2^ = .31, ε = .62) and high beta (F (5, 75) = 2.92, p = .006, η^2^ = .23, ε = .62) reflecting dynamic power changes across time. Additionally, for low alpha, a main effect of Plane (F (3, 45) = 4.369, p = .035, η^2^ = .23, ε = .49) reflected largest amplitudes over the parietal ROI. For theta, a main effect of Plane (F (3, 45) = 3.69, p = .029, η^2^ = .20, ε = .79) that was modified by a Time x Plane interaction (F (6, 90) = 3.32, p = .051, η^2^ = .18, ε = .33) was due to a power change over time for the frontal ROI across conditions (Time: (F (2, 30) = 4.88, p = .016, η^2^ = .25, ε = .94).

### Correlation between ERP Amplitude and ERSP Power

Pearson product moment correlation analysis revealed a significant positive relationship between late CNV amplitude and high alpha power over the frontal ROI in the 2600–3150 ms post-S1 time interval (r(16) = .67, p = 0.004) and the 3150–3700 ms time interval (r(16) = .70, p = .002) of Go-trials. Additionally, some correlations between middle or late CNV and alpha or beta showed a trend toward significance. These were the correlations between middle interval CNV and high alpha (frontal ROI, 1500–2050 ms post S1; r(16) = .55, p = 0.026 and 2050–2600 ms: r(16) = .59, p = 0.016), between late CNV and low alpha (frontal ROI, 2600–3150 ms: r(16) = .54, p = 0.032 and 3150–3700 ms: r(16) = .60, p = 0.014), between late CNV and low beta (frontal ROI, 2600–3150 ms: r(16) = .54, p = 0.029 and 3150–3700 ms: r(16) = .62, p = 0.011; central ROI, 2600–3150 ms: r(16) = .60, p = 0.013 and 3150–3700 ms: r(16) = .59, p = 0.016), and between late CNV and high beta (frontal ROI, 2600–3150 ms: r(16) = .54, p = 0.033).Neither theta nor gamma power correlated with CNV amplitude in any of the time intervals (p’s.091-.941).

## Discussion

We examined whether the CNV, an ERP index of preparatory brain activity, as well as event-related alpha power, showed differential activity in trials calling for preparation to respond to an upcoming stimulus compared to trials not requiring response preparation. Previous studies exploring alpha oscillatory activity preceding a Go/NoGo task versus no task have shown diverging results [29], [30]. We further assessed whether the hypothesized differential alpha Go/NoGo activity was accompanied by differential event-related activity in the theta, beta and gamma bands. Importantly, we report preparatory spectral activity in a Go/NoGo delayed response task where S1 consistently signaled whether or not a response was required to S2.

As observed previously [27], [28], the magnitude of the CNV response was enhanced following S1-Go relative to S1-NoGo signals (Figure 1B), suggesting that a Go-cue initiates more preparatory cortical activity than a NoGo cue. The CNV difference was accompanied by lower event-related alpha power in Go compared to NoGo trials (Figure 2B–C) over the investigated midline electrode groups. A difference in alpha power between conditions requiring varying degree of preparation is supported by one of two studies investigating alpha ERD during preparation for a Go/NoGo task versus no task [30]. In the one study that did not find an alpha power difference a non-significant difference was, however, observed in the last part of the S1–S2 interval [29]. In both studies S1 signaled whether the trial was a Go or NoGo trial. However, in Go trials only some of the S2s did indeed signal that the participant should make a response. Thus, motor preparation in Go trials was probably smaller than if Go trials had consistently called for motor preparation. The present study demonstrates that when Go and NoGo trials are sufficiently distinct, alpha power will reflect the difference in need for preparation. In the Babiloni et al. (2004) study task and no-task conditions were presented in separate blocks, thus demonstrating differential preparatory spectral activity between two separate tasks. The present study extends this result, showing that preparatory spectral activity is modulated on a trial-by-trial basis.

Importantly, the difference in spectral power between conditions was not only demonstrated for the alpha band alone, but for a broad band ranging from theta to low gamma (Figure 2 and S1). The gamma band was investigated as three individual subbands. In the low gamma band there was a significant Go/NoGo difference that reflected lower event-related power in the Go compared to the NoGo condition. In the 53–80 Hz gamma, an opposite effect, with higher power in the Go compared to the NoGo condition, was present only over the occipital ROI. However, an investigation of this power increase showed that it was caused by muscle activity in 3 participants. Indeed, scalp recorded gamma activity during a cognitive task can often be attributed to muscle activity [43], [44]. Thus, the higher 53–80 Hz gamma power in the Go compared to the NoGo condition over the occipital ROI likely does not reflect a difference in preparatory cognitive activity. The 81–100 Hz high gamma band did not show reliable differences between conditions and was also compromised by increased high-frequency muscle noise.

Gomez et al. found a generalized decrease in oscillatory activity ranging from delta to low gamma (0–42.9 Hz) during expectancy of a visual stimulus requiring a response [26]. In that study only Go trials were included. Broadband spectral activity has also been compared between trials requiring a response and trials not requiring a response. In an experiment designed to study response anticipation and response conflict Fan et al. investigated oscillations from 4 to 100 Hz [45]. They showed that response anticipation was associated with reduced theta, alpha and beta power in most of 9 dipoles (based on fMRI activations from the same task) and increased low and high gamma power in frontal and parietal dipoles. Response anticipation was measured as the difference in activity between trials where a “ready” cue was followed by a flanker task S2, and trials where a “relax” cue was not followed by the flanker task. Thus, the two conditions differed both in stimulus anticipation and response preparation, whereas in the present study stimulus anticipation was the same for both conditions. Accordingly, the gamma increase shown by Fan et al. could possibly be related to stimulus anticipation and reflect increased activation in networks relevant for stimulus processing. Indeed, it has been shown that enhanced prestimulus gamma, but not alpha, improves visual processing [46].

The present study and the studies mentioned above showed a decrease in delta to beta/gamma band power in the S1–S2 interval of Go- trials. In a study comparing “task” and “no task” trials, Molnar et al. (2008) found oscillatory modulations that were not uniform across bands, as there was no condition effect in the beta band, decreased power in the delta band and increased power in the alpha and theta bands in the “task” compared to the “no task” condition [47]. The lack of concurrence of results could be due to several methodological differences. Firstly, Molnar et al. performed a fast Fourier Transform on the entire time window and thus had no temporal resolution. In the present study main effects of time were found on both alpha bands. Secondly, Molnar et al. investigated one anterior and one posterior ROI, each comprising of mostly lateral electrodes. In the present study four midline ROIs, including central regions, were investigated. Differences in experimental designs might also contribute. The two conditions of the Molnar et al. study were presented in separate blocks, not interspersed with each other. Furthermore, only 40% of the trials in the block termed “task-trials” actually required a response, and this was defined by S2, not S1. Thus, in the majority of trials, the subjects did not expect to execute a response, consequently this was not a pure Go-condition. The alpha-increase in “task”-trials could therefore be related to response inhibition. Interestingly, Figure 2 shows that in the NoGo condition of the present study there was an increase in alpha activity that could also reflect preparatory inhibition of motor networks. Also, the weaker difference between “task” and “no-task” trials might contribute in explaining the lack of condition effects in the beta band.

The present study involved visual S2 stimuli. Studies investigating spectral activity during preparation for visual stimuli presented in attended versus non-attended locations have shown that alpha amplitude is increased in visual cortex ipsilateral to to-be attended locations and decreased contralaterally to to-be attended locations [48], [49]. The topoplots in Figure 2 show that in the present study there is no indication of hemisphere differences in spectral power. This can be explained by the lack of directional cues. First, the expected visual stimulus was presented centrally on the screen. Second, the motor response to the visual stimulus was executed with one or the other hand, but information about what hand should be used was delivered with S2. Thus, the required motor preparation was not lateralized. The differences in preparatory alpha activity between attended and ignored locations in the Worden et al. (2000) and Doesburg et al. (2009) studies could nevertheless reflect similar processes as in the Go/NoGo difference in alpha power of the present study. In our NoGo condition the information delivered by S2 could be ignored since it was not to be responded to, thus rendering this condition comparable to a non-attended condition. In this sense our paradigm is similar to those of studies investigating attended versus non-attended visual fields. In all of the three studies the ignore trials involved higher prestimulus alpha power than attend trials. This suggests that the alpha decrease in Go compared to NoGo trials in the present study could be related to differential anticipation for the visual stimulus, in addition to a difference in motor inhibition versus preparation. One study did, however, show that stimulus anticipation is not enough for alpha power to decrease in the preparatory interval. [50]. Go trial alpha power was decreased in the S1–S2 interval of trials where Go/NoGo information was delivered in S1 compared to trials where this information was delivered in S2. In the latter condition alpha ERD did not appear until after S2 was presented. Arguing that the S1–S2 interval of the first, but not the latter would involve motor preparation, the authors suggested that alpha ERD in the interstimulus interval is linked to motor preparation, supporting our hypothesis that an absolute difference in motor preparation will reveal alpha differences between Go and NoGo conditions.

In summary, the present study complements existing results, in showing that a broadband spectral Go/NoGo difference exist when the difference between conditions is restricted to motor preparation.

### Function of Spectral Go/NoGo Difference across Multiple Bands

Reduced alpha and beta power has been associated with increased cortical activation [51]. The reduced alpha and beta power in the Go condition of the present study thus suggests greater cortical activation when motor preparation is called for than when it is not. This would agree with a proposed mechanism of the CNV [1], [2] as a pre-activation of the cortical areas that will be needed to process and respond to the upcoming stimulus. Pre-activation would ensure a faster or more efficient processing of and response to S2. Indeed, decreased alpha activity in preparatory intervals has been shown to increase processing of the imperative stimulus [52]–[54]. We found that also theta and low gamma showed attenuated power in Go compared to NoGo trials. Whether theta and gamma power decreases reflect increased cortical activity in preparatory periods as well is uncertain. Regarding theta, an opposing pattern to that of alpha has been observed [55]. Klimesch showed that whereas alpha desynchronizes with increasing task demands, theta synchronizes. For gamma as well, an increase, rather than decrease, of power has been shown to correlate with the functional activation of the cortex [56]. Indeed, electrocorticography studies have shown increases in high frequency oscillations (60–200 Hz) in the presence of decreased power in lower bands [57], [58] and scalp EEG studies have shown simultaneous alpha ERD and low gamma ERS during movement [20], [59]. Furthermore, the attention orienting literature shows that whereas local alpha synchronization is associated with inhibition of not-to-be used networks [48], long-range gamma synchronization is associated with excitability of to-be-used networks [60] Low gamma has, on the other hand, also been reported to display power changes in the same direction as lower bands [26], [61]. Vijn et al. (1991) showed reduced EEG power in a spectral band ranging from 0.2 to 40 Hz during visual stimulation [61]. Moreover, as reported above, reduction of power from delta to low gamma has also been shown during expectancy periods [26]. In summary, the association between changes in alpha/beta versus theta/gamma is uncertain, but considerable evidence points to gamma and theta synchronization as a reflection of cortical activity. One interpretation of the power decrease in preparatory intervals, in all spectral bands from theta to gamma, could thus be that whereas the alpha and beta decreases reflect a preactivation of the neural networks that will be activated by the upcoming stimulus and response, the observed theta and gamma decreases could reflect inhibition of competing neural networks.

Rather than just reflecting preparation of the networks that will be recruited for processing of and responding to the imperative stimulus, and inhibition of competing networks, the reduction of power in several frequency bands could also reflect the different cognitive and motor processes that are more active in the S1–S2 interval of Go trials compared to NoGo trials. The most apparent difference between the two conditions of the present study is motor preparation, but the S1–S2 intervals of the two conditions are different in additional aspects. As the participants are told to always look at the center of the screen, where S2 will appear, both Go and NoGo trials will necessarily involve some degree of anticipation for the imperative stimulus. This anticipation could, however, be stronger in Go trials due to the higher task-relevance of S2. Also due to the stronger relevance of S2 in Go trials, sustained attention and working memory processes could be more strongly engaged in Go trials. Together, these cognitive processes have been associated with all of the frequency bands investigated in the present study. Alpha, beta and gamma have been associated with attention, alpha and beta with motor preparation, and alpha and theta with memory processes [24], [25]. A difference between conditions in the power of theta, alpha, beta and gamma could thus reflect a greater, or in the case of theta and gamma possibly reduced, involvement of brain processes associated with the different bands in the Go than in the NoGo condition. This does not, however, explain the similar development of power reduction over time across bands.

An alternative explanation for the similar activity in a broad spectral range, which could better account for the similar change in power over time, would be that the Go/NoGo difference in power reflects a difference in cortical excitability. In a review article Mathewson et al. advocated a role of alpha as a modulator of cortical excitability, with an appearance of low alpha power resulting in high cortical excitability and increased alpha power enabling low excitability. The authors left open the possibility that this mechanism might extend to other frequency oscillations [62]. In support of such a mechanism, Chen et al. used transcranial magnetic stimulation to show that cortical excitability increases during sensorimotor events that usually induce ERD of the 20 Hz rolandic rhythm and decreases after the sensorimotor events when rolandic rhythm ERS usually replaces ERD [63]. If the mechanism of alpha-oscillations as a modulator of cortical excitability extends to other frequency bands, an interpretation of the broadband reduction of power in Go compared to NoGo trials could be that it reflects increased excitability of neural networks in Go trials. This would ensure a higher probability for S2 to excite relevant networks in trials where this stimulus is important for behavior compared to trials where S2 does not have behavioral implications. Increased excitability in the different networks associated with a broad range of spectral oscillations may facilitate coordinated neural activity in distributed brain areas that will participate in the timely execution of the motor response at the occurrence of S2.

Cross-frequency coupling could be one mechanism enabling the timing of change in excitability modulated by the different frequency bands. Cross-frequency coupling has been shown in humans using scalp recordings [64], [65] as well as subdural electrodes [66], [67]. Oscillations in the neocortex tend to couple hierarchically [66], [68]. Lakatos et al. showed that delta band oscillations in the primary visual cortex entrain to the rhythm of a rhythmic stream of attended stimuli. Importantly, delta phase further determined momentary power in higher frequency activity (theta –gamma). Lakatos et al. suggested that the CNV could reflect resetting of a low frequency rhythm. In line with this, the power decrease across a wide range of frequencies observed in the present study could occur by co-modulation between low frequency oscillations and the higher frequency bands.

To summarize, we speculate that the broadband Go/NoGo difference in preparatory spectral activity observed in the present study could reflect increased excitability of neurons. It could also be related to an activation of neural networks relevant for processing of and responding to the upcoming stimulus, with a simultaneous inhibition of competing networks. The present study does not allow for a firm conclusion on the precise function of the broadband Go/NoGo difference, but both interpretations indicate that preparing involves extensive spectral changes that taken together increase the chance of efficient processing of and response to the upcoming signaled stimulus. The difference between conditions in a broad range of spectral oscillations demonstrates the importance of not only investigating a single predefined spectral band, but rather a wide spectral range to fully capture spectral changes associated with cognitive processes.

### Relation between ERP and ERSP

The present study demonstrates a significant difference between the activity in S1–S2 intervals of Go and NoGo trials for both ERP and theta to gamma ERSPs. Further, while the ERPs show a negative slow wave over frontocentral areas in Go trials, the ERSPs show a decrease of power. The central question then is whether the ERPs and ERSPs are a reflection of the same activity. There is an ongoing debate about whether ERPs are independent of background oscillatory activity or rather reflect phase resetting of ongoing brain oscillations [for a review, see 69]. Regardless of the mechanism underlying the generation of ERPs, ERSPs have been shown to provide additional information [12]. In addition to the time-and phase-locked activity revealed by the ERP, also phase-incoherent changes in the EEG spectrum are revealed in the ERSP. Studies investigating preparatory EEG activity support the notion that ERPs and spectral activity provide complementary information on preparatory brain processes [14], [15], [32], [70]. Recent work by Mazaheri and colleagues [13], [36] has, however, shown that asymmetric amplitude fluctuations of alpha oscillations can explain the generation of slow event-related responses. They found that modulations in the alpha, but not the beta band, explained their event-related field potentials, but noted that this mechanism might generalize to other frequency bands.

We investigated the relationship between ERP amplitude and ERSP power in Go trials over the two ROIs that showed a CNV; the frontal and the central regions. Positive correlations were found between late CNV and high alpha in the last two time intervals (2600–3700 ms), indicating that larger high alpha decrease was associated with larger CNV negativity. This leaves open the possibility that the late part of the CNV could be driven by alpha activity. Also, low alpha and beta power showed a trend towards positive correlations with CNV amplitude. Theta and gamma, on the other hand, did not correlate significantly with CNV amplitude. This could support the proposition that whereas the observed alpha and beta decrease reflect neural activation, as has also been proposed for the CNV, the theta and gamma decrease rather reflect inhibition of competing neural networks. An investigation of alpha amplitude asymmetry could provide further information on the relation between CNV and alpha, but is beyond the scope of this study. Regardless of the relationship between alpha, and possibly beta, and CNV, the present investigation of spectral activity provides complementary information on preparatory cortical activity to that of the ERP. The Go/NoGo difference across the theta to low gamma bands indicates coordinated preparatory activity in distributed neural networks.

### Conclusion

In conclusion, the present study extends previous findings by showing that Go versus NoGo cues presented in an event-related delayed response task induce not only differential modulation of the CNV, but also of power in a broad range of spectral oscillations, from theta to low gamma. Importantly, we demonstrate that the preparatory modulation of the spectral oscillations is similar across bands, with all bands showing a reduced power in Go compared to NoGo trials. This broadband power reduction could facilitate coordinated neural activity in distributed brain areas that participate in the timely execution of the motor response at the occurrence of the imperative stimulus.

## Supporting Information

Figure S1 **Broad-spectrum time-frequency plots for each condition and region of interest.** To visualize spectral activity across all bands, the same method was used as that for *a priori* band-specific analyses except for a broader range of frequencies. We used 35 log-spaced frequency bands between 4 and 100 Hz, calculated from the ERP data. The analytic amplitude (absolute value of the Hilbert transform) for each band was used to create grand average ERSP time-frequency plots for each region of interest, with the 1000 ms pre-S1 serving as baseline. Note that the Go/NoGo difference is evident across the spectrum, and not confined to specific frequency bands. Thus, the approach of choosing frequency bands a priori does not conceal frequency ranges not showing a Go/NoGo difference.
(TIFF)
