# Supplementary figures and images for: Differential Go/NoGo Activity in Both Contingent Negative Variation and Spectral Power
Source: PLoS One. 2012 Oct 31;7(10):e48504. doi: 10.1371/journal.pone.0048504 (PMC3485369; doi:10.1371/journal.pone.0048504)

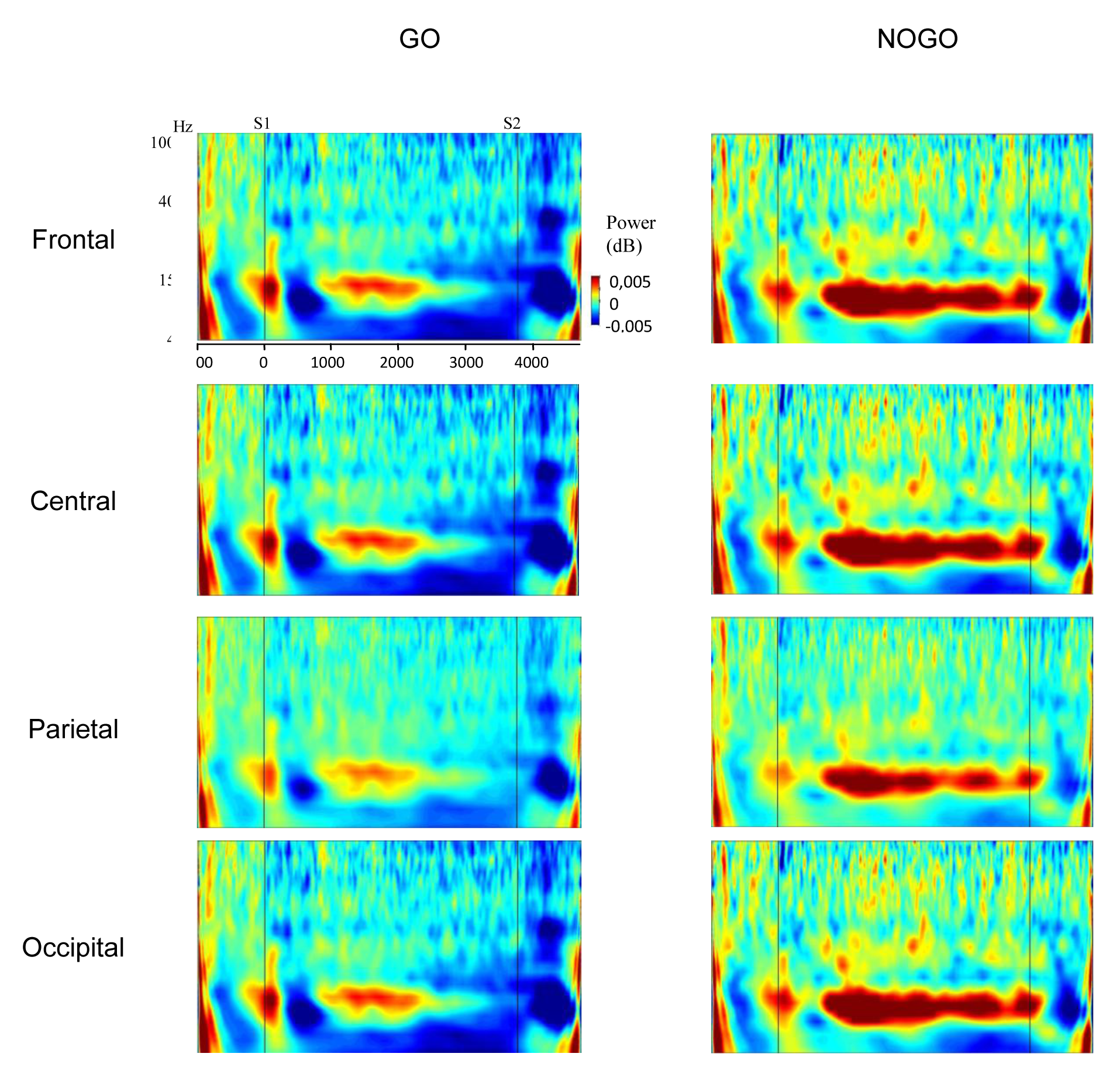

Supplement: Figure S1 — Broad-spectrum time-frequency plots for each condition and region of interest. To visualize spectral activity across all bands, the same method was used as that for a priori band-specific analyses except for a broader range of frequencies. We used 35 log-spaced frequency bands between 4 and 100 Hz, calculated from the ERP data. The analytic amplitude (absolute value of the Hilbert transform) for each band was used to create grand average ERSP time-frequency plots for each region of interest, with the 1000 ms pre-S1 serving as baseline. Note that the Go/NoGo difference is evident across the spectrum, and not confined to specific frequency bands. Thus, the approach of choosing frequency bands a priori does not conceal frequency ranges not showing a Go/NoGo difference. (TIFF) [file pone.0048504.s001.tif]
